# Femtosecond laser-induced nano- and microstructuring of Cu electrodes for CO_2_ electroreduction in acetonitrile medium

**DOI:** 10.1038/s41598-023-35869-z

**Published:** 2023-05-31

**Authors:** Iaroslav Gnilitskyi, Stefano Bellucci, Andrea Giacomo Marrani, Mariana Shepida, Artur Mazur, Galyna Zozulya, Vasyl Kordan, Volodymyr Babizhetskyy, Bouchta Sahraoui, Orest Kuntyi

**Affiliations:** 1 Lviv Polytechnic National University, 12 Bandery Str., Lviv, 79013 Ukraine; 2 “NoviNano Lab” LLC, 5 Pasternaka, Lviv, 79000 Ukraine; 3 INFN-Laboratori Nazionali di Frascati, Via E. Fermi 54, 00044 Frascati, Italy; 4 Dipartimento di Chimica, Università di Roma “La Sapienza”, p.le A. Moro 5, 00185 Rome, Italy; 5 Department of Inorganic Chemistry, Ivan Franko National University of Lviv, 6 Kyryla i Mefodiya Str., Lviv, 79005 Ukraine; 6 University of Angers, Photonics Laboratory of Angers LPhiA, SFR MATRIX, 2 Bd Lavoisier, 49045 Angers, France

**Keywords:** Ultrafast lasers, Catalysis, Environmental chemistry, Metals and alloys

## Abstract

The dependency of CO_2_ reduction rate in acetonitrile-Bu_4_NClO_4_ solution on cathodes, which were modified by laser induction of a copper surface, was studied. The topography of laser-induced periodic surface structures (LIPSS) → grooves → spikes was successively formed by a certain number of pulses. It was proved that for a higher number of laser pulses, the surface area of the copper cathode increases and preferred platy orientation of the copper surface on [022] crystallografic direction and larger fluence values increase. At the same time, the content of copper (I) oxide on the surface of the copper cathode increases. Also, the tendency to larger fluency values is observed. It promotes the increase of cathodic current density for CO_2_ reduction, which reaches values of 14 mA cm^-2^ for samples with spikes surface structures at E = − 3.0 V upon a stable process.

## Introduction

One of the main environmental problems at the planetary level is the increased concentration of CO_2_ in the atmosphere, which causes the greenhouse effect and the increase in the acidity of ocean and sea waters^1^. Taking into account the increasing trend of the concentration of this gas^2^, in recent decades, research has been actively conducted on reducing carbon(IV) oxide emissions and processing the latter into carbon-containing compounds. Electrochemical CO_2_ reduction is one of the promising routes of conversion of this gas into such valuable products: CO, CH_4_, C_2_H_4_, CH_3_OH, CH_3_COOH, CH_3_CHO, HCOOH, (COOH)_2_, etc.^3–6^. Reactions of formation of these products upon cathodic polarization in aqueous solutions (1–4) are characterized by relatively close values of standard electrode potentials^6^. This causes the low selectivity of CO_2_ conversion for any product. In aqueous solutions at E^0^ = − 0.83 V (vs. NHE) the electrochemical reduction of water begins (5), the share of which increases with increasing cathode potential, which limits the value of cathode potentials to − 1.0… − 1.3 V. In addition, the solubility of CO_2_ in aqueous solutions is low, which causes concentration polarization.

1 $${\text{CO}}{}_{2} + 6{\text{H}}_{2} {\text{O}} + 8{\text{e}}^{ - } = {\text{CH}}_{4} + 8{\text{OH}}^{ - } ,\;{\text{E}}^{0} = - 0.66\;{\text{V}}$$

2 $${\text{CO}}_{{2}} + {\text{ 5H}}_{{2}} {\text{O }} + {\text{ 6e}}^{ - } \to {\text{ CH}}_{{3}} {\text{OH }} + {\text{ 6OH}}^{ - } ,\;{\text{E}}^{0} = \, - 0.{\text{81 V}}$$

3 $${\text{CO}}_{{2}} + {\text{ H}}_{{2}} {\text{O }} + {\text{ 2e}}^{ - } \to {\text{ CO }} + {\text{ 2OH}}^{ - } , \;{\text{E}}^{0} = \, - 0.{\text{93 V}}$$

4 $${\text{CO}}_{{2}} + {\text{ H}}_{{2}} {\text{O }} + {\text{ 2e}}^{ - } \to {\text{ HCOO}}^{ - } + {\text{ OH}}^{ - } , \;{\text{E}}^{0} = \, - {1}.0{\text{8 V}}$$

5 $$2{\text{H}}_{2} {\text{O}} + 2{\text{e}}^{ - } \to {\text{H}}_{2} + 2{\text{OH}}^{ - } ,\;{\text{E}}^{0} = - 0.83\;{\text{V}}$$

Electrochemical reduction of CO_2_ in nonaqueous medium, primarily in ionic liquids^7–9^ and organic aprotic solvents^10–16^, makes it possible to eliminate or reduce the mentioned disadvantages of aqueous solutions. In the absence of water, CO_2_ is converted into oxalate anion (6, 7) and CO (8)^12,17^. Therefore, they are the main products in the environment of organic aprotic solvents^16^. Moreover, their high electrochemical stability makes it possible to reduce CO_2_ even at cathodic potentials up to − 3.5 V without side reactions^18–20^. In addition, the solubility of CO_2_ in organic aprotic solvents is one order of magnitude larger than its solubility in water. It achieves high i_cathode_ values up to 80 mA cm^−2^ and faradaic efficiencies (FEs) of up to 80%^12^.

6 $${\text{CO}}_{{2}} + {\text{ e}}^{ - } \to \cdot{\text{CO}}_{{2}}{^{( - )}}$$

7 $${\text{CO}}_{{2}}{^{( - )}} + \cdot{\text{CO}}_{{2}}{^{( - )}} \to \, \left( {{\text{COO}}} \right)_{{2}}{^{2 - }}$$

8 $$2{\text{CO}}_{2} + 2{\text{e}}^{ - } \to {\text{CO}} + {\text{CO}}_{3}{^{2 - } }$$

Electrochemical reduction of CO_2_ is a catalytic process, so the rate of conversion in aqueous solutions^3–5,21–23^ and organic aprotic solvents^18^ depends on the nature of the cathode surface and structure.

In recent years, enhanced attention has been paid to the electrode topography influence on the electrochemical processes of CO_2_ conversion and, accordingly, on the yield of products^10,12,23–30^. The most studied ones in this regard are copper cathodes, of which high efficiency is shown by those with high surface roughness^24–27^, foam-like structure^27^, highly porous 3D skeletons (sponges)^28^, and dendritic formations^29^.

Laser treatment is one of the newest promising methods of forming a highly developed surface for catalytically active CO_2_ reduction electrodes^26,27^. Laser treatment has been demonstrated as an efficient technology to induce micrometric structures over the surface of semiconductors^31,32^, metals^33–35^, dielectrics^36^ and polymers^37^. Not long time ago, the technique of laser-induced periodic surface structures (LIPSS), known for its high regularity, has made significant advancements due to its ability to achieve nanometer uniformity and its single-step, maskless process with industrial production speed^38^. Many studies have showcased the diverse applications of LIPSS, such as in holography^39^, surface-enhanced Raman spectroscopy (SERS)^40^, tribology^41^, sensors^42^, plasmonics^43^, and others^44,45^. By finely adjusting different parameters, the use of ultrashort laser pulses enables the creation of a broad range of microstructures with complex configurations. By varying the number of laser pulses and adjusting the laser fluence, one can generate hexagons, grooves, and spikes^46,47^. This approach demonstrates the unparalleled versatility of ultrashort lasers, making them applicable to nearly any manufacturing process. Moreover, this single-step process does not require a vacuum or other complex setups^44,45^.

Recently, the fabrication of LIPSS has been tested for secondary electron emission reduction on copper samples using linearly and circularly polarized femtosecond laser pulses, reporting on the influence of the formed surface textures on the secondary electron yield (SEY), thus addressing the possible role on the secondary electron yield of LIPSS on a copper surface with subwavelength-sized features^48^. The use of a cylindrical lens in femtosecond laser surface structuring also received attention, with aim of improving the processing efficiency, suitable for large area processing with circular and elliptical laser beams^49^.

The present work aims to establish the dependency of the rate of CO_2_ reduction in an organic aprotic solvent (acetonitrile) on the topography of a copper cathode modified by femtosecond laser pulses.

## Results and discussion

### Laser-induced surface modification

**Figure 1:**
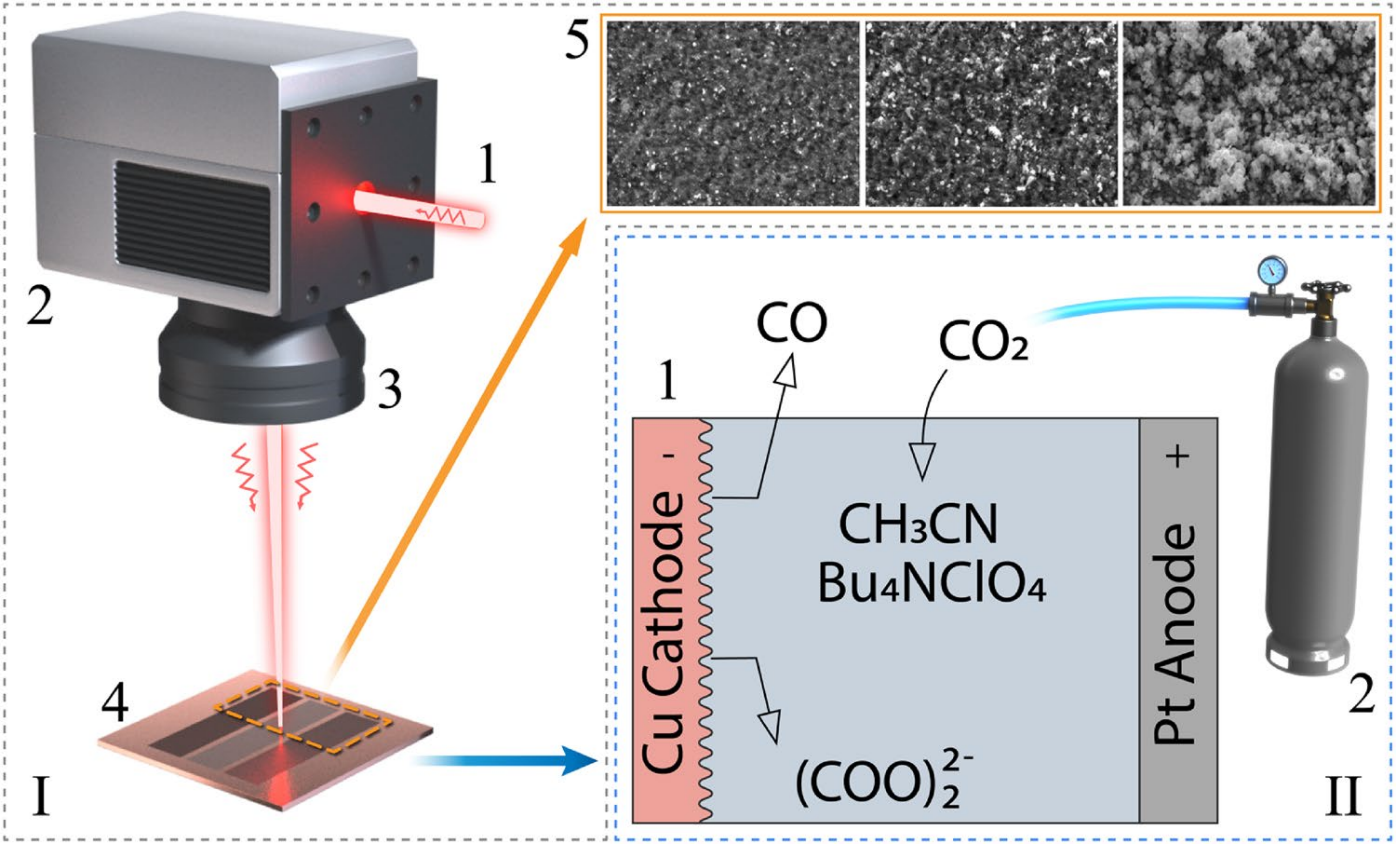
Sketch of experiments which consists of a laser setup for generation of LIPSS (I) and block of electrochemical conversion (II). Laser setup involves: 1—femtosecond laser beam, 2—galvoscanner, 3—F-theta lens, 4—Cu sample, 5—SEM images recorded from irradiated samples. Electrochemical conversion scheme consists of: laser treated samples of Cu as cathode in electrolyzer (1). CO_2_ gas supplies from balloon (2). The figure was created by using Adope Illustrator^50^.

Experimental workflow for CO_2_ reduction in electrochemical conversion scheme with cathodes that modified by femtosecond laser pulses presented on Fig. 1.

**Figure 2:**
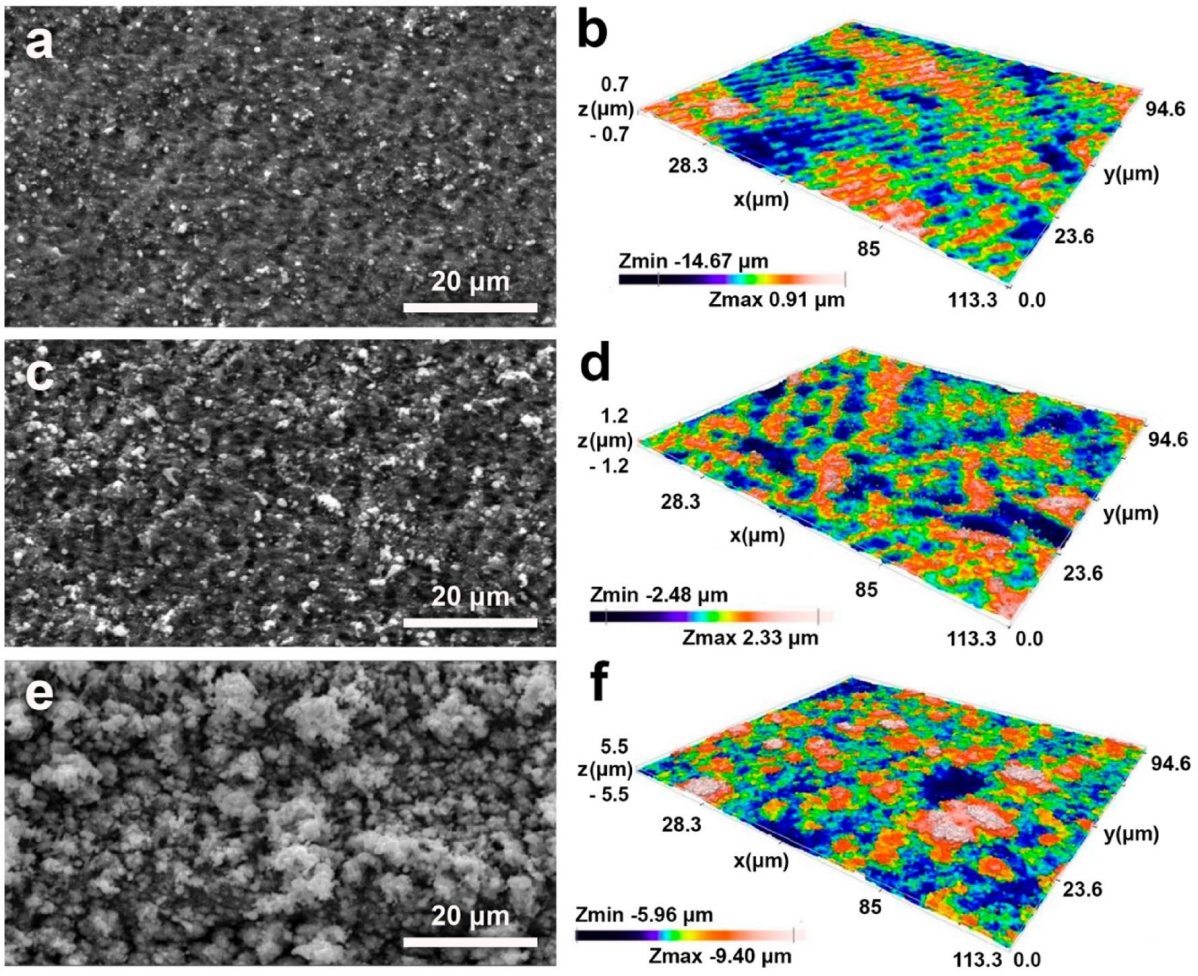
Left panel: SEM images of femtosecond laser-induced periodic surface structures of copper surface manufacturing: (**a**) LIPSS; (**c**) grooves; (**e**) spikes. Right panel: 3D-profiles of the laser modified surfaces: (**b**) LIPSS; (**d**) grooves; 3-D profiles were created by using Sensofar Metrology (Version 6.7.4.0)^51^.

Laser treatment results in the formation of *periodic surface structures* (Fig. 2) that homogeneously cover a large area (1 cm^2^). Figure 2a shows the sample denominated “LIPSS”. Figure 2c displays the sample “grooves” with structures that are preferentially aligned along a direction parallel to the laser polarization, and their generation usually occurs at larger fluence values and for higher number of laser pulses, with respect to ripples. Figure 2e shows the “spikes” sample, displaying self-organized structures that have spherical form in the micrometer scale generated upon polarized ultrashort pulses, with energy per pulse well above the ablation threshold. Another condition to form spikes is the high repetition rate to maintain the heat accumulation process. Such heat accumulation results in complex hydrodynamic processes, as it was also suggested in the paper^45^. The consequence is an increase in the surface dispersion with the formation of structures resembling the cauliflower (Fig. 2e). As shown in^26^, the latter is characterized by porous hierarchical structures, which is one of the conditions for increasing the copper cathode catalytic activity in CO_2_ conversion^22–29^.

3D-profiles of the laser modified surface measured by laser profilometer (LP) is presented in Fig. 2 (right panel). The root mean square roughness of the non-treated surface (R_a_) amounts to 57 ± 5 nm according to the LP images. The surface of the “LIPSS” sample (Fig. 2b) has a R_a_ of 70.4 ± 20 nm, while the “grooves” and “spikes” surfaces display a R_a_ of 118 ± 20 nm and 319 ± 20 nm, respectively (Fig. 2d,f). In all types of self-organised nano-microstructures the surface structures appear to be homogeneously distributed.

### XRD measurements

**Figure 3:**
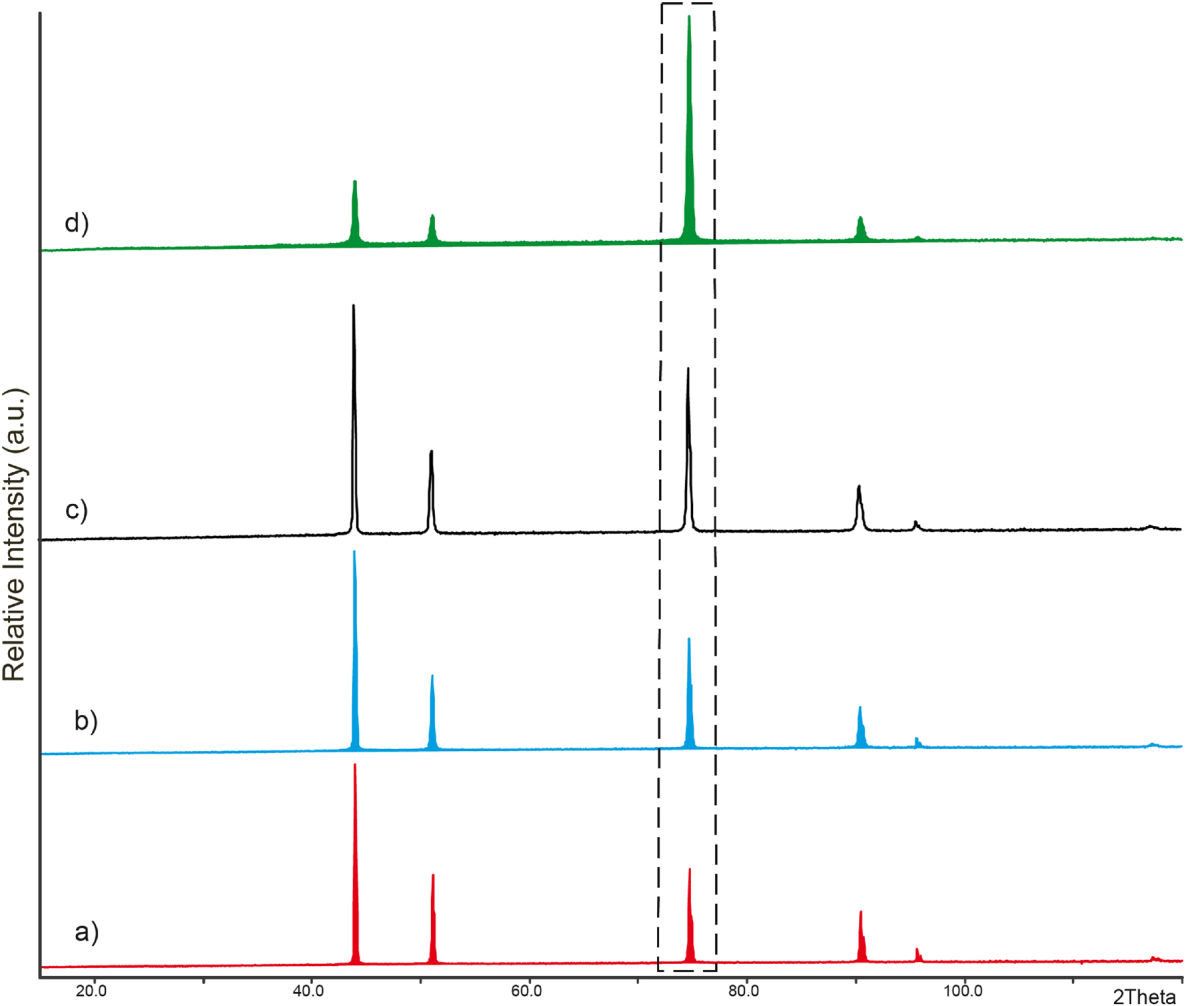
XRD patterns of untreated copper plate (**a**) and femtosecond laser-treated copper surface: (**b**) LIPSS, (**c**) grooves, (**d**) spikes. [002] peak is highlighted by dashed line.

In order to determine the lattice parameters and possible crystal structure transformations on the surface, the whole pattern fitting of Rietveld refinement was applied to the acquired XRD data. Figure 3 shows XRD patterns for different laser-treated copper surfaces. All the XRD patterns correspond to cubic copper phase. The results of refinement show nearly no changes of lattice parameters, a = 3.615 Å. They correspond to the literature data^52^. No shift of the peaks was observed.

The difference between XRD patterns was only in the relative intensities of peaks. The XRD pattern of the copper plate with LIPPS structures is less changed in comparison to the untreated copper XRD pattern. The peak intensity assigned to the [022] plane increases significantly, indicating the increment of textured effects of the copper surface. The refined coefficients of the preferred orientation using the March-Dollase function^53^ are 0.520(1) for untreated Cu, 0.503(1)—LIPPS, 0.458(1)—grooves and 0.319(1)—spikes. The decrease of the values indicates the increase of the amount of the preferred platy orientation for the treated copper plate surface. It agrees with the augmentation of larger fluence values and for higher number of laser pulses.

### XPS measurements

**Figure 4:**
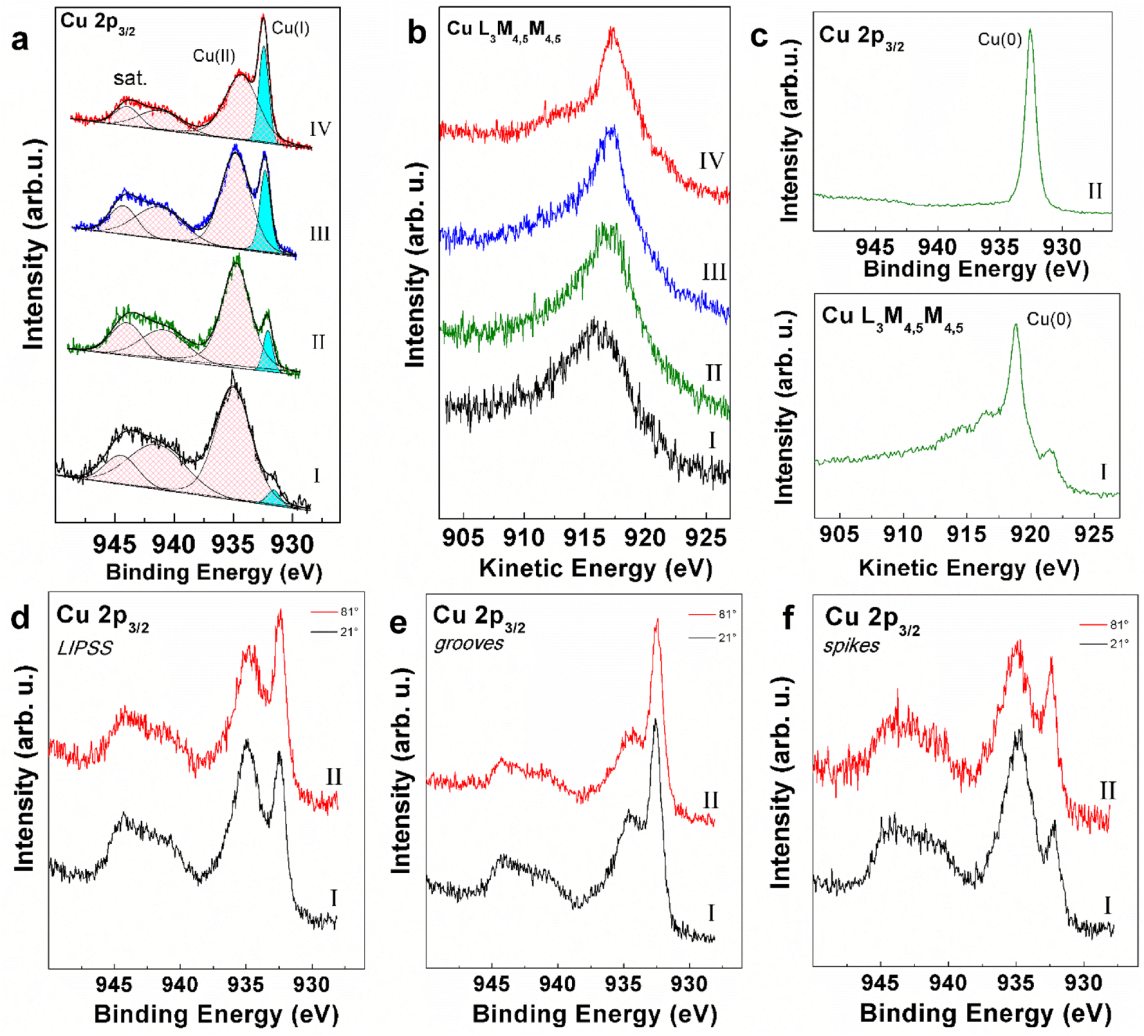
(**a**) Cu 2p3/2 XPS regions of (I) untreated (black), (II) spikes (green), (III) LIPSS (blue), and (IV) grooves (red) samples with curve-fitting results. (**b**) Cu L3M4,5M4,5 Auger spectra of samples in the same sequence. (**c**) Cu 2p3/2 XPS (I) and Cu L3M4,5M4,5 Auger (II) spectra of a reference pure Cu(0) sample. (**d**–**f**) Comparison between Cu 2p3/2 XPS regions acquired at photoelectron take-off angles of (I) 21° and (II) 81° for the (**d**) LIPSS, (**e**) grooves and (**f**) spikes samples.

XPS is known to be the election technique for surface chemical analysis, given the short inelastic mean free path of excited photoelectrons, and the high sensitivity of their kinetic energy towards the atomic and molecular structure of the investigated system. XPS analysis was conducted on all laser-treated samples (LIPSS, spikes and grooves), and on a reference untreated copper sample. The large photoionization cross-section region of Cu 2p was recorded (see Figs. S1, S2 and S3 for further regions), and the corresponding *j* = 3/2 spin–orbit component is shown in Fig. 4a for each sample after curve-fitting deconvolution, at the photoelectron take-off angle of 21°. The spectrum of untreated copper surface (Fig. 4a,I) is dominated by a broad component (pink-shaded) at 934.9 eV binding energy (BE) followed by a satellite extending in the range 940–945 eV. In copper compounds this latter feature is attributable to an unscreened core-ionized final state at high BE, split due to core-valence spin coupling, which is typical of *d*^*9*^ (Cu^2+^) species^54^. The presence of this feature coupled to the position of the main peak calls for the presence of a layer of Cu(II) oxide on the surface of the untreated copper sample.

Stepping to the laser-treated samples (Fig. 4a,II–IV), it is apparent the growth of a low-energy component at 932.2 eV, which was already slightly outlined in spectrum I of Fig. 4a. This component increases in intensity in the sequence of samples: untreated < spikes < LIPSS < grooves (I < II < III < IV, respectively), at the expenses of the broad peak at 934.9 eV. According to its BE position, this peak could be associated to the presence of both Cu(0) and Cu(I), which are known to be indistinguishable in terms of Cu 2p BE^54,55^. Accordingly, the Cu L_3_M_4,5_M_4,5_ X-ray excited Auger electron spectrum was acquired for all the samples (see Fig. 4b), and an additional Cu(0) metal reference sample was analyzed in both Cu 2p and Auger regions (see Fig. 4c). The Auger electron spectra display a typical Cu(II) lineshape in the case of untreated sample (Fig. 4b,I), confirming the Cu 2p spectrum interpretation, which smoothly turns into that of a mixed Cu(II)/Cu(I) species for the other samples (Fig. 4b,II–IV)^56^. Comparison with the reference Auger spectrum of copper metal (Fig. 4c,I) rules out the detection of Cu(0) in all the Cu 2p spectra (Fig. 4a), likely due to the presence of a layer of native copper oxide onto the electrodes surface. The Cu(II)/Cu(I) ratio was quantified via curve fitting from the areas of the corresponding components (pink-shaded and cyan-colored peaks, respectively), considering that the core-valence coupling derived satellite is attributable solely to Cu(II). The obtained ratios are 34.6, 14.9, 5.4 and 3.8 in the sequence of samples: untreated > spikes > LIPSS > grooves (I > II > III > IV, respectively), supporting a decrease of Cu(II) in favor of Cu(I) from the untreated to the grooves sample.

In order to investigate the depth distribution of the Cu(I) component detected in the laser-treated electrodes, XPS spectra were acquired also at grazing (81°) photoelectron take-off angle, which implies a shorter surface sampling depth (Fig. 4d–f). A comparison for each sample at the two take-off angles shows that the Cu(I) signal is enhanced at grazing angle, pointing at the presence of Cu(I) species as a surface overlayer, with the Cu(II) layer just below it.

Furthermore, according to Fig. 4a, the Cu(I) enrichment occurs only in the laser-treated samples and to a different extent for each of them. In the recent past, it has been demonstrated that laser treatment of Cu(II) oxide (CuO) leads to chemical reduction and formation of cuprous Cu(I) oxide (Cu_2_O) and eventually copper metal, according to the adopted experimental conditions^56^.

It is probable that also in this case a similar mechanism is active, i.e. a laser-induced reduction of native CuO onto the copper electrode according to reaction (9). Among the investigated samples, “grooves” is the one with the most prominent Cu(I) component, which could be due to the synergistic effect of high laser energy and high repetition rate. In the works^24,57,58^ showed that in aqueous solutions, Cu_2_O on the surface of copper electrodes catalyzes the reduction of CO_2_ to CO at low overpotentials. This helps to increase the values of i_cathode_ with high faradaic efficiencies. A similar effect of Cu_2_O should also be expected for CO_2_ electroreduction in organic aprotic solvents.

9 $${\text{Cu}} + {\text{CuO}}\xrightarrow{t}{\text{Cu}}_{2} {\text{O}}$$

### Electro-chemical measurements

**Figure 5:**
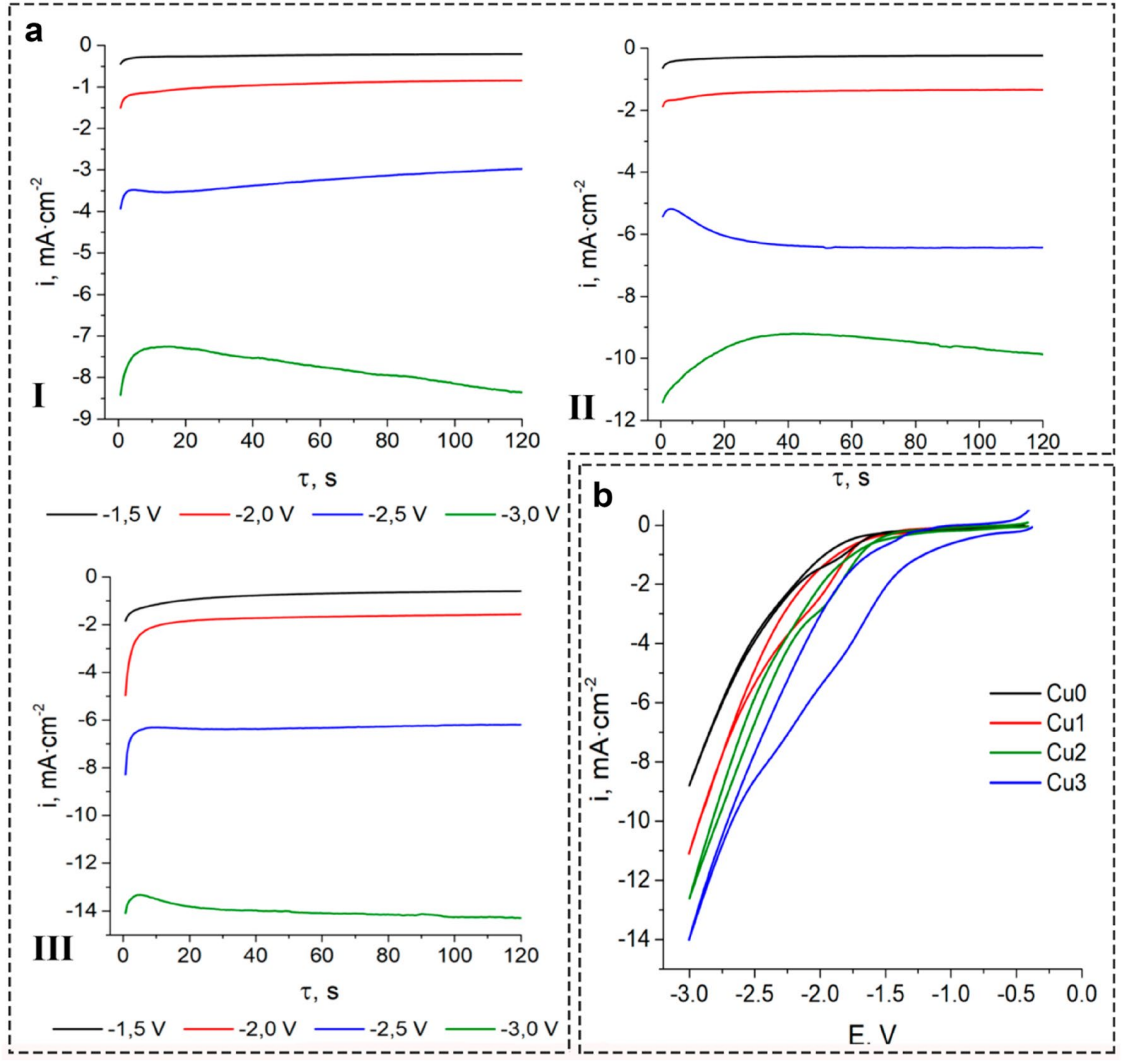
(**a**) Potentiostatic electrolysis on copper electrodes in 0.05 M CO_2_-saturated Bu_4_NClO_4_ acetonitrile solutions: Cu1—LIPSS; Cu2—grooves; Cu3—spikes. (**b**) Cyclic voltammograms for Cu electrode in 0.05 M CO_2_-saturated Bu_4_NClO_4_ acetonitrile solutions: (I) Cu0—untreated; (II) Cu1—LIPSS; (III) Cu2—grooves; (4) Cu3—spikes.

Cyclic voltammograms (CVs) for Cu electrodes in CO_2_-saturated acetonitrile solutions (Fig. 5a) are typical for the environment of organic aprotic solvents^10–13,18,59^. Appreciable values of cathodic currents are observed at E < − 2 V. This is due to the non-aqueous medium factor, where in the absence of water, the prevailing cathodic reaction (6) takes place, the value of the standard electrode potential of which is low. It has been reported that in organic aprotic solvent DMF E^0^CO_2_/CO_2_ = − 2.21^59^ or − 1.97 V^60^ vs. SHE. It should be expected that in acetonitrile solutions, this value is approximately the same.

The copper surface topography effect is manifested in the values of the cathodic currents from the beginning of the active electrochemical reduction of CO_2_ (E ~ − 1.5 V) to E = − 3.0 V (Fig. 5b(II)). At copper cathodes with spikes, i_cathode_ almost twice the value of this value prevails at cathodes from a smooth copper surface. Moreover, the current values do not decrease over time (Fig. 5a), which indicates the stability of the electrochemical reduction of CO_2_ at the modified copper cathodes.

In addition to the increase in the specific area of the copper electrode, the cathodic currents growth is obvious due to the increase in surface activity. It is especially evident over electrodes with spikes, which are characterized by an increased concentration of active centers. The topography of the surface causes the aforementioned (Fig. 2e) and it also causes larger fluence values, which are occurring by an increase of the amount of preferred platy orientation for the treated copper plate surface (Fig. 5).

With a cathode potential increase, a rapid rise in the values of the cathode currents is observed (Fig. 5b). It can be explained by the influence factor of the organic aprotic solvent. Acetonitrile, as a polar molecule, is adsorbed on a copper cathode with the formation of surface complexes due to the donor–acceptor interaction Cu ← :N≡C–CH_3_. Consequently, the cathode surface is blocked. An increase in cathodic potentials, i.e., an increase in negative charge, causes the desorption of CH_3_CN, which facilitates the processes of reduction by reactions (6, 8).

The rate of electrochemical reduction of CO_2_ on the surface of femtosecond laser modification cathode is almost equal to the rate of reduction on the gold cathode, and the value of i_cathode_ is second only to the Pb gas diffusion electrode (Table 1). However, the latter is technologically difficult to manufacture.

**Table 1:** Conditions for the electrochemical reduction of CO_2_ in the organic aprotic solvents.

Cathode	Electrolyte	E, V	i_cathode_, mA cm^−2^	Main CO_2_ conversion products	Ref
Cu on basal Pt(hkl) single crystal faces	TBAPF_6_ in AN or PC	− 1.8	0.88 0.88 in AN, 0.28 in PC	CO	^10^
Au foil	Bu_4_NClO_4_ in PC	− 2.8	16	CO	^11^
Pb gas diffusion electrode	NEt_4_BF_4_ in AN	− 2.5	80	oxalic acid	^12^
Cu, Pt, Au and Pb discs	0.1 M TEABF_4_ (46 ppm H_2_O)	− 2.4	0.5	CO	^13^
Bulk gallium	Bu_4_NCl, Bu_4_NBr, Bu_4_NI and Bu_4_NClO_4_ in DMSO, DMF, NMP, PC and AN	–	–	CO	^19^
Au foil	Catholyte: 0.1 M Bu_4_NClO_4_ in PC	− 3.0	2.8	CO	^20^
Nanoporous TiO_2_	0.1 M Bu_4_NClO_4_ in dry AN	− 1.8	0.8	CO + oxalate	^15^
WO_3_ nanoparticulate thin films on FTO glass	0.1 M Bu_4_NClO_4_ in dry AN (10 ppm H_2_O)	− 1.2	0.3	CO	^21^
Femtosecond laser modification	0.05 M Bu_4_NClO_4_ in AN	− 3.0	14	CO + oxalate	This work

## Conclusions

In contrast to aqueous solutions, CO_2_ reduction in organic aprotic solvents allows for the electrolysis in a wide range of cathode potentials (up to E = − 3.0 V), yielding the carbon(II) oxide and oxalate without side processes. This feature of the non-aqueous medium was used to study the efficiency of the surface modification of copper cathodes by laser induction. As the number of pulses and the intensity of laser processing increase, three types of copper surface topography LIPSS → grooves → spikes are successively formed. As a result, the cathode area, preferred platy orientation of the copper surface on [022] crystallographic direction and larger fluence values increase. At the same time, the content of copper (I) oxide on the surface of the copper cathode increases, which increases its electrocatalytic activity. It contributes to increasing CO_2_ reduction currents, from 8 mA cm^−2^ for LIPSS cathodes to 14 mA cm^−2^ for spikes at E_constant_ = -3.0 V, which is identical to an increase in the rate of cathodic conversion of carbon(IV) oxide.

## Materials and methods

### Manufacturing of femtosecond laser-induced periodic surface structures (LIPSS), grooves and spikes over the copper foil surface

Laser irradiation was carried out on electrolytic copper foil with the use of an Yb:KGW laser source operating at a wavelength of 1030 nm. The laser emitted linearly polarized pulses with a pulse duration of 266 fs.

In order to control the movement of the laser beam, a galvanometric scanning head (ExceliScan, ScanLab) equipped with an F-theta lens was employed. The focal distance of the lens was set to 72 mm.

The samples were fixed on a computer-controlled 6-axis translational stage (Standa, Lithuania). The spot size was determined to be approximately 11.5 μm in diameter at 1/e^2^ intensity. Laser parameters were outlined in Table 2.

**Table 2:** Laser parameters for Cu surface modification.

No	Power, W	Energy, μJ	RR, kHz	Step, μm	Speed, m/s	Number of pulses	Fluence, J/cm^2^
LIPSS	2.2	4.4	500	4.5	1	5.75	1.059
Grooves	4	8	500	4.5	1	5.75	1.926
Spikes	4	11	333	5	0.18	21.28	2.648

### Cathode reduction of CO_2_ on copper surface in acetonitrile solution

The electrochemical reduction of CO_2_ on copper cathodes with femtosecond laser-induced periodic surface structures was studied by cyclic voltammetry and chronoamperometry in a 0.05 M tetrabutylammonium perchlorate (Bu_4_NClO_4_) solution of acetonitrile (CH_3_CN, AN). The solution was pre-saturated with carbon dioxide for 30 min. For research was used a standard three-electrode electrochemical 50 cm^3^ cell, a working 1 × 1 cm electrode, a platinum auxiliary, and a silver chloride reference electrode (Ag/AgCl, E^0^ = 0.198 V vs. NHE, all potential values will henceforth be referred to this electrode). Cyclic voltammograms for copper electrode were performed within the potential range from E = 0.0 V to − 3.0 V with a potential sweep speed of 50 mV s^−1^. Potentiostatic electrolysis was carried out at E = − 1.5; − 2.0; − 2.5; − 3.0 V. Electrochemical studies were carried out using an MTech PGP-550S potentiostat.

### Morphological study

SEM examination of the samples was carried out by using an electron microscope Tescan Vega 3 LMU equipped with an X-MaxN 20 silicon drift detector. Overall compositions were investigated using energy-dispersive X-ray spectroscopy (EDX); Gun voltage 25 kV, shooting mode SE- and BSE-detectors, working distance 15–16 mm, vacuum 10^–3^ Pa.

### Phase analysis by XRD

The phase analysis of four specimens was performed using X-ray diffraction data. The XRD intensity data were collected on an automatic diffractometer HZG-4a (CuKα radiation, λ = 1.54179 Å, 2θmax = 120°, step-scan mode with a step size of 0.05°(2θ) and a counting time of 25–30 s per data point, Si calibration external standard). In this study, the WinCSD program package^61^ was used for quantification and calculation of structural parameters.

### X-ray photoelectron spectroscopy (XPS) analysis

XP spectra were recorded using a modified Omicron NanoTechnology MXPS system equipped with a monochromatic source (Omicron XM-1000) and an Omicron EA-125 energy analyzer. The exciting radiation used was Al Kα (*hυ* = 1486.7 eV), generated operating the anode at 14 kV and 16 mA. All photoionization regions were acquired using an analyzer pass energy of 20 eV, except for the survey scan, taken at 50 eV pass energy. Take-off angles (*θ*) of 21° and 81° with respect to the sample surface normal were adopted, with the latter corresponding to a thinner sampling depth (higher surface sensitivity). The measurements were performed at room temperature, and the base pressure in the analyzer chamber was about 2 × 10^–9^ mbar. Experimental data were fitted using a linear function to reproduce the secondary electrons’ background and pseudo-Voigt functions for the elastic peaks. These curves are described by a common set of parameters (position, FWHM, Gaussian–Lorentzian ratio) which were let free to vary within narrow limits. The Gaussian–Lorentzian ratio was left free to vary between 0.7 and 0.9. Experimentally determined area ratios (with ± 10% associated error) were used to estimate XPS atomic ratios between Cu(II) and Cu(I) components.

## Supplementary Information

Supplementary Figures.

## Data Availability

The datasets used and analysed during the current study available from the corresponding author on reasonable request.
