# Supplementary material for: Femtosecond laser-induced nano- and microstructuring of Cu electrodes for CO2 electroreduction in acetonitrile medium
Source: Sci Rep. 2023 May 31;13:8837. doi: 10.1038/s41598-023-35869-z (PMC10232451; doi:10.1038/s41598-023-35869-z)
Supplement: Supplementary file 1 — Supplementary Figures. [file 41598_2023_35869_MOESM1_ESM.docx]

**Femtosecond laser-induced nano- and microstructuring of Cu electrodes for CO_2_ electroreduction in acetonitrile medium**

Iaroslav Gnilitskyi^a,b,c^, Stefano Bellucci^c *^, Andrea Giacomo Marrani^d^, Mariana Shepida^a^, Artur Mazur^a^, Galyna Zozulya^a^, Vasyl Kordan^c^, Volodymyr Babizhetskyy^e^, Bouchta Sahraoui^f^ and Orest Kuntyi^a^

*^a^* Lviv Polytechnic National University, 12 Bandery Str., 79013, Lviv, Ukraine

*^b^* “NoviNano Lab” LLC, 5 Pasternaka, 79000, Lviv, Ukraine

*^c^* INFN-Laboratori Nazionali di Frascati, Via E. Fermi 54, 00044 Frascati, Italy

*^d^* Dipartimento di Chimica, Università di Roma “La Sapienza”, p.le A. Moro 5, I-00185, Rome, Italy

*^e^* Department of Inorganic Chemistry, Ivan Franko National University of Lviv, 6 Kyryla i Mefodiya Str., 79005 Lviv, Ukrainef

*^f^* University of Angers, Photonics Laboratory of Angers LPHIA, SFR MATRIX, 2 Bd Lavoisier, 49045 Angers, France

*Corresponding author.

*E-mail address:* iaroslav.gnilitskyi@novinano.com

SUPPORTING INFORMATION





**Figure S1**. XPS survey spectra of (I) untreated, (II) spikes, (III) LIPSS, and (IV) grooves samples.





**Figure S2**. XPS C 1s spectra of (I) untreated, (II) spikes, (III) LIPSS, and (IV) grooves samples.





**Figure S3**. XPS O 1s spectra of (I) untreated, (II) spikes, (III) LIPSS, and (IV) grooves samples.
